# Heterozygous truncating variants in *SUFU* cause congenital ocular motor apraxia

**DOI:** 10.1038/s41436-020-00979-w

**Published:** 2020-10-07

**Authors:** Simone Schröder, Yun Li, Gökhan Yigit, Janine Altmüller, Ingrid Bader, Andrea Bevot, Saskia Biskup, Steffi Dreha-Kulaczewski, G. Christoph Korenke, Raimund Kottke, Johannes A. Mayr, Martin Preisel, Sandra P. Toelle, Sarah Wente-Schulz, Saskia B. Wortmann, Heidi Hahn, Eugen Boltshauser, Anja Uhmann, Bernd Wollnik, Knut Brockmann

**Affiliations:** 1 Interdisciplinary Pediatric Center for Children with Developmental Disabilities and Severe Chronic Disorders, Department of Pediatrics and Adolescent Medicine, University Medical Center, Göttingen, Germany; 2 Institute of Human Genetics, University Medical Center, Göttingen, Germany; 3 Cologne Center for Genomics, Center for Molecular Medicine Cologne, University of Cologne, Cologne, Germany; 4 Department of Clinical Genetics, University Children’s Hospital, Paracelsus Medical University, Salzburg, Austria; 5 Department of Pediatric Neurology, University Hospital Tübingen, Tübingen, Germany; 6 Praxis für Humangenetik Tübingen, Tübingen, Germany; 7 Department of Pediatric Neurology, University Hospital Oldenburg, Oldenburg, Germany; 8 Department of Diagnostic Imaging, University Children’s Hospital, Zurich, Switzerland; 9 Department of Pediatrics, University Hospital Salzburg, Paracelsus Medical University, Salzburg, Austria; 10 Department of Pediatric Neurology, University Children’s Hospital, Zurich, Switzerland; 11 Department of Pediatric Kidney, Liver and Metabolic Diseases, Hannover Medical School Children’s Hospital, Hannover, Germany; 12 Radboud Center for Mitochondrial Medicine, Department of Pediatrics, Amalia Children’s Hospital, Radboudumc, Nijmegen, The Netherlands; 13 Cluster of Excellence “Multiscale Bioimaging: from Molecular Machines to Networks of Excitable Cells” (MBExC), University of Göttingen, Göttingen, Germany

**Keywords:** SUFU, congenital ocular motor apraxia, COMA, sonic hedgehog, Joubert syndrome

## Abstract

**Purpose:**

This study aimed to delineate the genetic basis of congenital ocular motor apraxia (COMA) in patients not otherwise classifiable.

**Methods:**

We compiled clinical and neuroimaging data of individuals from six unrelated families with distinct clinical features of COMA who do not share common diagnostic characteristics of Joubert syndrome or other known genetic conditions associated with COMA. We used exome sequencing to identify pathogenic variants and functional studies in patient-derived fibroblasts.

**Results:**

In 15 individuals, we detected familial as well as de novo heterozygous truncating causative variants in the Suppressor of Fused (*SUFU*) gene, a negative regulator of the Hedgehog (HH) signaling pathway. Functional studies showed no differences in cilia occurrence, morphology, or localization of ciliary proteins, such as smoothened. However, analysis of expression of HH signaling target genes detected a significant increase in the general signaling activity in COMA patient–derived fibroblasts compared with control cells. We observed higher basal HH signaling activity resulting in increased basal expression levels of *GLI1*, *GLI2*, *GLI3*, and *Patched1*. Neuroimaging revealed subtle cerebellar changes, but no full-blown molar tooth sign.

**Conclusion:**

Taken together, our data imply that the clinical phenotype associated with heterozygous truncating germline variants in *SUFU* is a *forme fruste* of Joubert syndrome.

## INTRODUCTION

The term congenital ocular motor apraxia (COMA), introduced by Cogan in 1952, designates the inability to initiate saccades, i.e., the eye movements performing rapid gaze shift. In his original report, Cogan described four children with a distinct disturbance of voluntary horizontal gaze characterized by the “inability to turn the eyes voluntarily in a direction for which there is full involuntary…control” accompanied by compensatory, jerky head movements.^1^ COMA usually affects horizontal, but rarely also vertical saccades.

Ocular motor apraxia (OMA) is observed in a wide range of conditions.^2^ A frequent and consistent co-occurrence of early-onset (congenital) OMA, also designated infantile-onset saccade initiation delay, with early-onset cerebellar ataxia and global developmental delay was emphasized.^3–9^ Most of the patients described experienced gradual resolution of OMA and ataxia over their first decade of life, whereas cognitive impairment persisted to a variable extent.^4,7–9^ These reports shaped a concept of COMA as a clinical entity and likely inherited as an autosomal recessive disorder, although Cogan, in his original report, described COMA as a symptom, not a diagnosis. However, no gene associated with isolated COMA (OMIM 257550) has been identified yet.

Of note, this condition is sometimes also called Cogan syndrome type 2, thus distinguishing it from Cogan syndrome type 1, a rare inflammatory disorder characterized by interstitial keratitis and sensorineural hearing loss, occasionally accompanied by systemic vasculitis.

More recently, doubts have arisen “whether Cogan-type oculomotor apraxia can exist as an isolated entity.”^10^ Liu and coworkers^11^ assigned COMA to three major clinical conditions: (1) benign or idiopathic variant with normal neuroimaging and only occasionally occurring neurologic symptoms; (2) nonprogressive, noninherited variant with structural brain anomaly caused, e.g., by dysgenesis of the cerebellar vermis or corpus callosum, inferior vermian hypoplasia, Dandy–Walker malformation, gray matter heterotopias, and perinatal ischemia; and (3) “part of a genetic syndrome” variant, which includes, e.g., Joubert syndrome, Jeune syndrome, and a subset of patients with Leber congenital amaurosis.

In a previous study aimed at a nosological delineation of COMA, we investigated a cohort of 21 patients diagnosed as having COMA.^12^ In that study, a reappraisal of neuroimaging revealed a molar tooth sign (MTS) indicative of Joubert syndrome in 11 and specific neuroimaging features pointing to other diagnoses in 2 patients. The remaining eight subjects had normal magnetic resonance image (MRI) or nonconclusive neuroimaging features, which led to a descriptive diagnostic classification of COMA.^12^

Pursued investigation of these eight patients and recruitment of further subjects with COMA framed the study presented here. We report six unrelated families comprising 15 affected individuals with distinct clinical features of COMA who do not share common diagnostic characteristics of Joubert syndrome or other known genetic conditions. We identified heterozygous truncating germline variants in the Suppressor of Fused (*SUFU*) gene and were able to show de novo occurrence of these *SUFU* variants in two families and inheritance from a mildly affected parent in the remaining four families. On a cellular level, COMA patient–derived fibroblasts show a generally high basal Hedgehog (HH) signaling activity, which, however, can be regulated by exogenous HH signaling activator or inhibitor treatment.

## MATERIALS AND METHODS

### Subjects

We compiled clinical data of 15 subjects from six unrelated families. Clinical phenotypes and MRI data of three subjects were included in our previous report.^12^ An additional six subjects with heterozygous *SUFU* variants and, in most cases, early-onset ocular apraxia were recruited from the families of these three index patients. Six further subjects, five of them with COMA, from three families were recruited through national and international collaborations with the attending neurologists.

### Ethics statement

All studies were performed in accordance with the Declaration of Helsinki protocols. The studies were reviewed and approved by the local institutional ethics board (University Medical Center Göttingen, Göttingen, Germany; file numbers 19/5/14 and 3/9/14). Written informed consent was obtained from all affected subjects, parents, or legal representatives participating in this study.

### Clinical and qualitative neuroimaging analysis

Data on developmental course and neurological features were collected by review of the clinical histories and by clinical–neurological follow-up investigations. Information about ophthalmological features was compiled from neuro-ophthalmological or pediatric neurological reports. Cognitive function was assessed using standardized neuropsychological tests whenever possible, or it was appraised from the patient’s history, clinical examination, and school reports. Additional information was gathered in telephone interviews with the patients or their parents using a standardized questionnaire.

Neuroimaging data were available for ten subjects. Two subjects received a new MRI in line with this study for technically optimized assessment of the hindbrain. All available MRI data sets were qualitatively analyzed by two pediatric neurologists with experience in neuroimaging of the brainstem and cerebellum. All imaging sequences in axial, coronal, and sagittal orientation were scrutinized with a focus on size and position of the superior cerebellar peduncles, hypo-/dysplasia of the cerebellar vermis, cerebellar cysts, brainstem morphology including shape of the interpeduncular fossa at the section of the brainstem isthmus and upper pons, size and shape of the 4th ventricle, and any other cerebellar or cerebral malformations, as described previously.^12^

### Exome sequencing and variant screening

In families 1, 2, 3, and 6,^13^ trio-based exome sequencing (ES) was performed. In family 4, a next-generation sequencing (NGS) panel of 13 genes associated with ciliopathies was applied. All detected *SUFU* variants were confirmed by polymerase chain reaction (PCR) amplification and subsequent Sanger sequencing on an independent DNA sample and tested for cosegregation within the respective families. In the affected subject II.1 of family 5, all exons and adjacent exon–intron boundaries of *SUFU* were analyzed by PCR and subsequent, bidirectional Sanger sequencing in a candidate gene approach.

Details of ES and variant screening as well as methods of cell culture and treatments, analysis of cilia formation, immunofluorescence staining, real-time quantitative PCR, and statistical analysis are provided as Supplementary Information.

## RESULTS

### Clinical and neuroimaging phenotypes of individuals with COMA

**Fig. 1:**
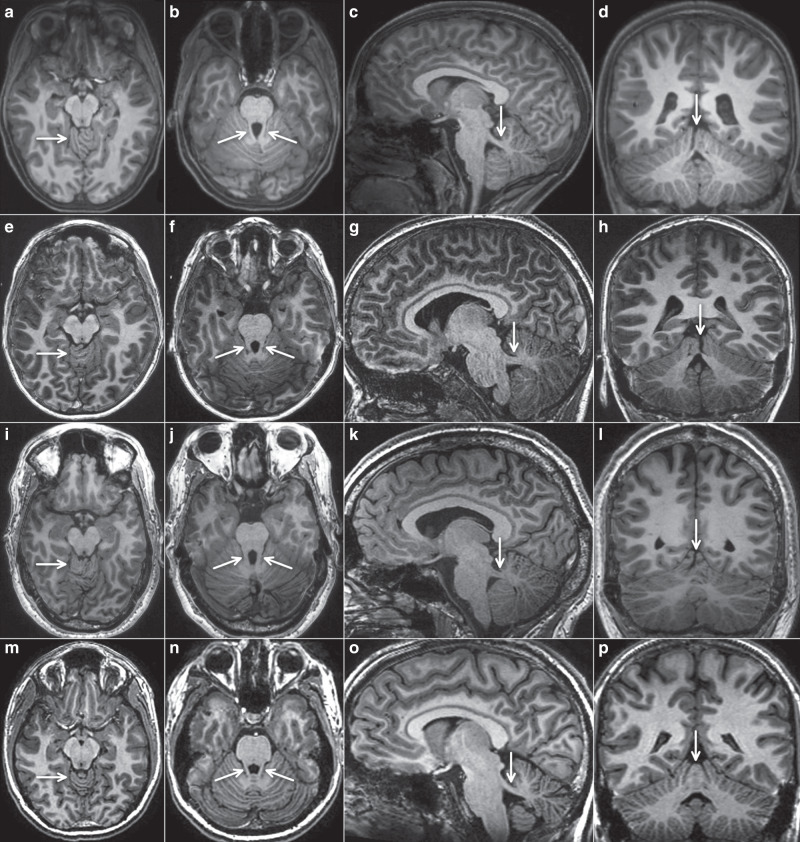
Characteristic magnetic resonance image (MRI) features in three subjects with heterozygous *SUFU* variants. Four representative T1-weighted MRIs (arranged in horizontal rows) are shown from three individuals with *SUFU* variants and one adult healthy control (**m**–**p**). Panels (**a**–**d**) are from subject I.1, family 3, at age 1.5 years; (**e**–**h**) from subject II.1, family 2, at age 7.5 years; and (**i**–**l**) from subject II.1, family 5, at age 40 years. The first vertical row (**a**,**e**,**i**,**m**) shows axial views at the level of the upper vermis, indicating folial dysplasia (arrow). The second vertical row (**b**,**f**,**j**,**n**) illustrates axial views at the level of the superior cerebellar peduncles (arrows), these are more prominent (longer, thicker) compared with normal. The third vertical row (**c**,**g**,**k**,**o**) shows parasagittal sections demonstrating that the superior cerebellar peduncles (arrows) are thicker and have a more horizontal course compared with normal (**o**). The fourth vertical row (**d**,**h**,**l**,**p**) illustrates coronal images revealing irregular folia and vermis splitting (arrows) in the individuals with *SUFU* variants.

Through national and international collaborations, we recruited 15 individuals from six families with a clinical diagnosis of COMA and without conclusive neuroimaging features, notably without definite molar tooth sign. Three of these patients, III.6 (family 1), II.1 (family 2), and II.1 (family 3), were presented in a previous clinical study without any genetic findings.^12^ Detailed clinical information of affected individuals is summarized in Table 1. Brain MRI was available from ten subjects including four from a parent, and was deemed conspicuous in families 3 through 6 already at first evaluation. Standardized qualitative reanalysis of all MRI data sets revealed strikingly similar neuroimaging features in all subjects. Figure 1 illustrates the relevant findings from three individuals in comparison with a healthy control. We did not observe any signs of a classical, full-blown molar tooth sign. However, the superior cerebellar peduncles were abnormally prominent in all cases, thickened, elongated, and had a more horizontal course, best seen in parasagittal sections. On axial sections through the upper vermis, a cerebellar folia dysplasia was obvious in eight cases and present in a milder form in two individuals. Coronal views were available in seven subjects and revealed an upper vermis split in all seven cases. The interpeduncular fossa had a normal appearance. The fourth ventricle had a normal shape and, on midsagittal view, the fastigium was not cranially displaced. Supratentorial anomalies were not discernible (Fig. 1). Of note, vermis folia dysplasia and upper vermis split are typical findings in Joubert syndrome.^14^

**Table 1:** Clinical and genetic features of 15 individuals with heterozygous *SUFU* variants.

Family # (origin)	Patient # (# in Wente et al.^12^)	Sex	Current age (years)	*SUFU* variant		Development		Neurological findings				MRI of the brain available
				cDNA position	AA change	Unaided walking at age (months)	Speech delay	Ocular apraxia (onset, course)	Early-onset ataxia	Cognitive development	Head circumference at last follow-up (SD)	
1 (T)	I:2	F	64	c.83C>A	p.Ser28*	11	Yes	No	n.a.	n.a.	- 0.6	No
	II:3	F	41	c.83C>A	p.Ser28*	“Normal”	Yes	No	No	Learning disability	- 0.2	No
	II:5	F	37	c.83C>A	p.Ser28*	10	No	No	No	Normal	+ 0.2	No
	II:8	M	21	c.83C>A	p.Ser28*	32	Yes	5 months, ↓	Yes	Learning disability	+ 1.3	Yes
	III:3	M	6	c.83C>A	p.Ser28*	20	Yes	5 months, ↓	Yes	Delayed	+ 1.0	No
	III:6 (10)	M	13	c.83C>A	p.Ser28*	24	Yes	6 months, ↓	Yes	Learning disability	+ 0.1	Yes
2 (S/CH)	I:1	M	42	c.1099G>T	p.Glu367*	27	No	No	Yes	Normal	+ 1.3	Yes
	II:1 (14)	M	11	c.1099G>T	p.Glu367*	24	Yes	6 months, ↓	Yes	Normal	+ 0.9	Yes
3 (D)	II:1 (19)	F	10	c.479delA (de novo)	p.His160Leufs*20	14	No	6 months, ↓	No	Normal	+ 2.6	Yes
4 (AL)	II:1	M	2	c.1220_1221ins T (de novo)	p.Phe408Valfs*13	21	No	8 months, ↓	Yes	Normal	- 0.2	Yes
5 (CH)	I:1	M	40	c.309_310delAG	p.Arg103Serfs*3	“Late”	No	Early childhood, still present	No	Normal	+ 3.0	Yes
	II:1	M	6	c.309_310delAG	p.Arg103Serfs*3	22	Yes	6 months, ↓	Yes	Normal	+ 0.6	Yes
6 (A)	I:2	F	37	c.[1333dupG]	p.[Glu445Glyfs*22]	24	No	No	No	Normal	+ 1.3	Yes
	II:1	M	5	c.[1333dupG]	p.[Glu445Glyfs*22]	19	No	6 months, ↓	Yes	Normal	+ 1.8	Yes
	II:2	M	2	c.[1333dupG]	p.[Glu445Glyfs*22]	21	Yes	10 months, =	Yes	n. a.	+ 2.1	No

*A* Austrian origin, *AA* amino acid, *AL* Albanian origin, *cDNA* complementary DNA, *CH* Swiss origin, *D* German origin, *MRI* magnetic resonance image, *MTS* molar tooth sign, *n. a.* not available, *OA* ocular apraxia, *S* Sardinian origin, *SCP* superior cerebellar peduncles, *SD* standard deviation, *T* Turkish origin, ↓ attenuating, = unchanged.

### Family 1

Family 1 is a multigenerational family with several individuals showing clinical features of OMA. Individual II.8 was diagnosed with early-onset OMA at the age of five months. During childhood, ataxia, global developmental delay, and learning disability became apparent. His maternal half-sister, individual II.3, had delayed motor and speech development. She attended a special school due to learning disability. She reports no head jerks or problems with horizontal pursuit, and her relative recalled no signs of OMA during her childhood. Her son (III.3) was diagnosed with OMA at the age of five months. He presented with ataxia, delay in motor and speech development as well as cognitive impairment. Individual II.5, the sister of II.3, had normal motor, speech, and cognitive development. Early OMA is not recalled by other, older family members. She attended regular school, is now a housewife and mother, and has no vocational training. Her son, individual III.6, presented with early-onset OMA, ataxia, motor and speech developmental delay as well as cognitive impairment. Clinical data for individual I.2, the mother of II.3, II.5, and II.8, was limited, but delay in speech development was reported during early childhood.

### Family 2

Individual II.1 was diagnosed with early-onset OMA, ataxia, and delay in motor and speech development (Fig. 1e–h). His cognitive performance is normal. His father, individual I.1, was allegedly healthy. However, clinical re-evaluation and consultation of the paternal grandmother revealed motor developmental delay with impaired balance in his first years of life. He walked without support at two years three months and had poor motor coordination throughout kindergarten age. Abnormal eye movements or head jerks were not noted. He learned “very late” to ride a bicycle and tie his shoes. At school, he easily learned reading, but was not able to write properly. Over the years, these symptoms ameliorated and he was unimpaired as an adult.

### Family 3

Individual II.1 had early-onset OMA, but normal motor, speech, and cognitive development. Mild balance problems were observed in her first years of life, but overt ataxia was not reported in repeated neurological examinations over the years (Fig. 1a–d). She presented with secondary macrocephaly with a head circumference of +2.6 SD at last follow-up at age nine years, while the head circumferences of both parents are normal. She had surgical excision of a digital tumor at age six years. Histopathological investigation of the excised tissue revealed a single fibroma without evidence of malignancy. There are no additional clinical signs pointing toward Gorlin–Goltz syndrome (OMIM 109400) in this patient.

### Family 4

Individual II.1 presented with early-onset COMA. The boy showed mild motor developmental delay, muscular hypotonia, and early-onset ataxia. His speech development is currently normal and his cognitive development seems to be normal, but assessment is unsecure at age of only 2 years. Clinical evaluation of his parents did not reveal any sign of OMA.

### Family 5

Individual II.1 presented with early-onset OMA, ataxia, and motor as well as speech development delay. As his neuroimaging features showed overlap with patterns observed in other patients of this study, direct testing of *SUFU* was initiated (Fig. 1i–l). His father, individual I.1, had a longstanding history of ophthalmological treatment including several surgical interventions for strabismus. His mother used to urge him to practice horizontal gaze pursuit, and he is accustomed to his jerky head movements. Currently, he shows mild impairment of horizontal saccades and occasional head jerks. A diagnosis of OMA was not established previously, however, and onset of OMA cannot be determined. His cognitive development was normal and he accomplished an academic career.

### Family 6

Individual II.1 and his younger brother II.2 were both diagnosed with early-onset OMA at the age of 6 and 10 months, respectively. Both had early-onset ataxia, muscular hypotonia, and mild motor as well as speech developmental delay. Neuroimaging features of individual II.1 resembled the pattern observed in other patients in this study. A heterozygous *SUFU* variant was identified using ES. Clinical history of their mother, individual I.2, revealed unaided walking at two years of age, mainly due to sickle feet. Besides this, her motor and cognitive development was normal and OMA was not reported in childhood nor present in adulthood.

### Identification of heterozygous truncating *SUFU* variants

**Fig. 2:**
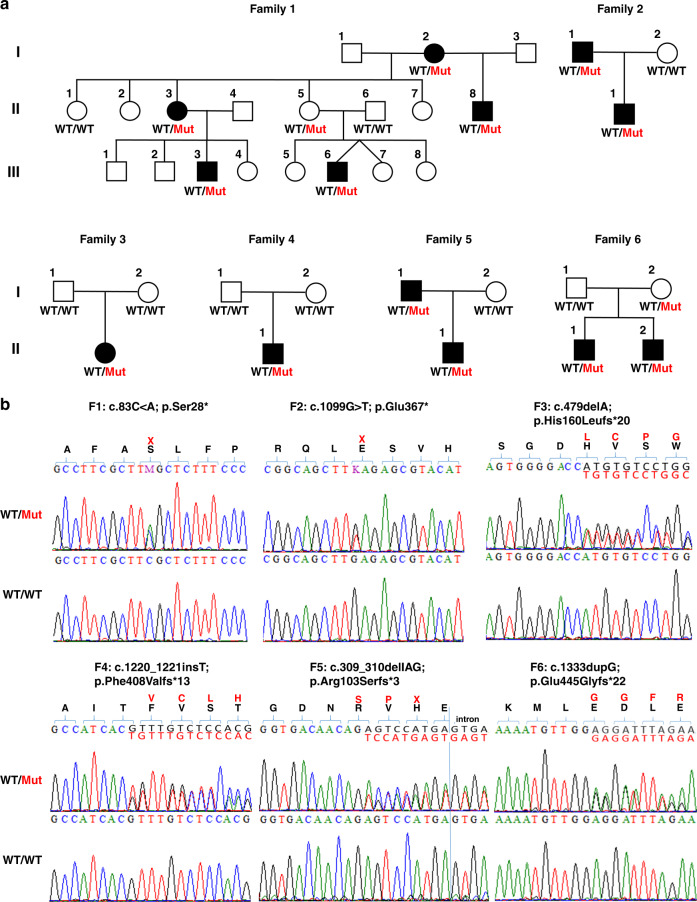
Pedigrees and genetic characterization of six families with congenital ocular motor apraxia carrying heterozygous loss-of-function variants in *SUFU*. (**a**) Pedigrees of families 1–6 showing segregation of rare deleterious *SUFU* variants. Unfilled shapes denote healthy, filled shapes affected individuals. (**b**) Chromatograms of the identified *SUFU* variants in family 1 (F1: c.83C>A; p.Ser28*), family 2 (F2: c.1099G>T; p.Glu367*), family 3 (F3: c.479delA; p.His160Leufs*20), family 4 (F4: c.1220_1221insT; p.Phe408Valfs*13), family 5 (F5: c.309_310delAG; p.Arg103Serfs*3), and family 6 (F6: c.1333dupG; p.Glu445Glyfs*22) compared with wild-type (WT) sequences of the respective positions. Localization of frameshift or nonsense variants is indicated in red.

To identify the underlying genetic cause of COMA, we performed ES of affected individuals from families 1, 2, 3, 4, and 6 and their parents. ES data were filtered for de novo, homozygous, or compound heterozygous variants with a coverage of more than six reads, a minimum quality score of 10, an allele frequency ≥25%, and an minor allele frequency (MAF) <0.5% in the gnomAD database.^15^ Based on the similar clinical presentation, we filtered for variants in overlapping genes in all affected individuals in these families, and we were able to identify variants in only a single overlapping gene in all five families. All affected individuals carried heterozygous truncating variants in the *SUFU* gene (Fig. 2).

In individuals II.1 (family 3) and II.1 (family 4) we identified the heterozygous truncating variants c.479delA (p.His160Leufs*20) and c.1220_1221insT (p.Phe408Valfs*13) in *SUFU*, respectively, and we confirmed the de novo status of these variants in the affected individuals. Individuals III.3 (family 1), II.1 (family 2), and II.1 and II.2 (family 6) inherited *SUFU* variants from one of their parents, and clinical re-evaluation revealed mild to moderate clinical symptoms in individual II.3 (family 1), I.1 (family 2), as well as I.2 (family 6). In individual II.1 (family 5), we analyzed all exons and exon–intron boundaries in *SUFU* by Sanger sequencing in a candidate gene approach based on the characteristic MRI features. We identified a heterozygous frameshift variant, c.309_310delAG (p.Arg103Serfs*3), in *SUFU* that was inherited by the affected father.

**Fig. 3:**
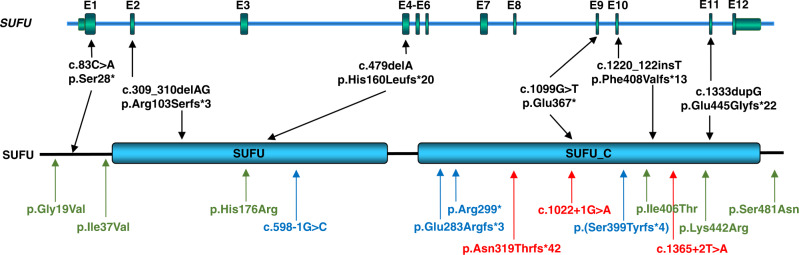
Overview of the identified heterozygous loss-of-function variants in *SUFU* on genomic and protein level. Schematic diagram of the *SUFU* gene (top panel) and protein (bottom panel) showing the localization of six truncating variants identified within this study in affected individuals from six independent families (black). Localization of *SUFU* variants associated in previous studies with Joubert syndrome are indicated in green and blue; variants associated with Gorlin–Goltz syndrome are marked in red.^19,22,27,28^.

All six variants were not observed in any current database of human genetic variations including gnomAD (access date 26 April 2020), and they are predicted to lead to SUFU protein truncation (Fig. 3). SUFU is a highly conserved protein that is under strict mutational constraint. In gnomAD, which contains the genetic data of more than 250,000 alleles, only 194 missense variants were observed while 286 were expected for *SUFU*, resulting in a *z*-score of 1.93. Moreover, no homozygous truncating variants were observed in *SUFU* and only three heterozygous truncating variants were detected in gnomAD (probability of loss of function intolerance [pLI] score of 1), indicating that *SUFU* is extremely intolerant to loss-of-function variants.

### *SUFU* variants lead to impaired repression of HH signaling signature genes

**Fig. 4:**
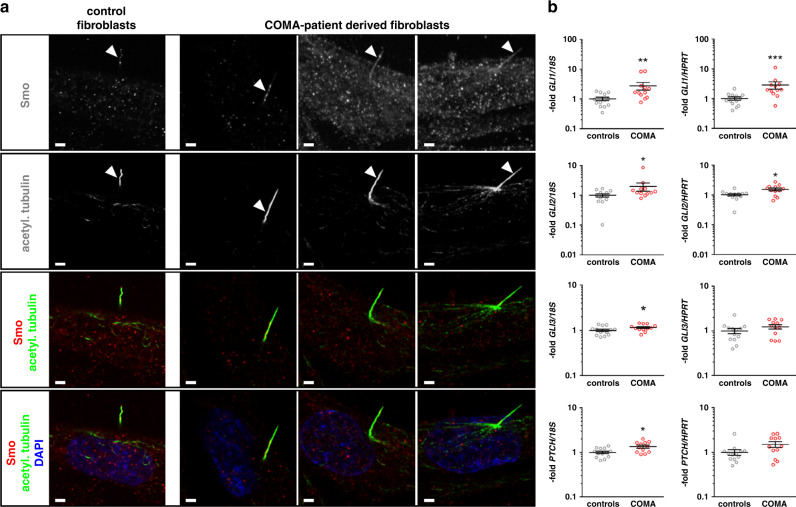
Cilia formation and expression of Hedgehog signaling signature genes in congenital ocular motor apraxia (COMA) patient–derived dermal fibroblasts. (**a**) Representative pictures of double immunofluorescent staining for visualization of SMO (upper row in monochrome, third and lower row in red) and acetylated tubulin (acetyl.tubulin) (second row in monochrome, third and lower row in green) in control fibroblasts and fibroblasts derived from affected individual II.2 (family 3, first column), individual II.1 (family 2, middle column), and individual II.1 (family 4, right column). Nuclei were visualized by DAPI staining (lower row in blue). Scale bars: 1 µm. (**b**) Quantitative real-time polymerase chain reaction (PCR)–based expression analyses of the Hedgehog signaling signature genes *GLI1*, *GLI2, GLI3*, and *PTCH1* normalized to *18S* ribosomal RNA (rRNA) (left column) or *HPRT* (right column) expression levels, respectively, of controls (*N* = 5) and COMA patient–derived fibroblasts (COMA) (*N* = 4). Shown results represent data of two different cellular passages per fibroblast culture each analyzed in biological triplicates (gray circles) that were measured in technical triplicates. Total mean values +/- SEM of all analyzed samples are indicated in black. Significant differences were tested by nonparametric Mann–Whitney tests. *p < 0.05; **p < 0.01; ***p < 0.001. No significantly different expression levels were observed in the five independent control fibroblast cultures, different cellular passages, or biological triplicates. For comparison of individual gene expression levels of the four independent COMA patient–derived fibroblast cultures as well as *HIP* expression levels see Supplemental Data (Fig. S1).

SUFU is a negative regulator of the HH signaling pathway. In the absence of active HH signaling, SUFU binds to cytosolic GLI proteins, thereby restricting their activity and inducing their truncation, which in turn promotes GLI repressor formation and induces repression of their target gene expression. Since the existence of intact primary cilia is required for HH signaling in vertebrates, we first analyzed whether patient-derived fibroblasts form acetylated tubulin positive cilia. However, we did not observe any differences in cilia occurrence, morphology, or ciliary localization of SMO in patient-derived fibroblasts compared with wild-type cells, indicating that the identified truncating variants in *SUFU* have no impact on this organelle essential for HH signaling (Fig. 4a). To assess whether the *SUFU* variants have an effect on the repressor function of SUFU, we analyzed the expression of HH signaling target genes in wild-type and COMA patient–derived dermal fibroblasts by quantitative real-time PCR (Fig. 4b and S1). Interestingly, we observed a significant increase in the general activity in COMA patient–derived fibroblasts compared with control cells resulting in higher basal expression levels of *GLI1*, *GLI2*, *GLI3*, and *PTCH1* (Fig. 4b). Treatment of cells with the HH signaling agonist SAG led to a further increase in target gene expression in both wild-type and patient-derived fibroblasts, with higher expression levels in patient-derived cells (Fig. S1). Similarly, inhibition of HH signaling using the Smoothened inhibitor vismodegib downregulates HH signaling and target gene expression to respective basal levels, still resulting in higher activity in vismodegib-treated patient fibroblasts compared with wild-type fibroblasts (Fig. S1). Nevertheless, the extent of HH signaling regulation upon SAG and/or vismodegib treatment did not differ between COMA patient–derived and control fibroblasts (Fig. S1). Overall, these results suggest that the identified truncation variants in SUFU lead to higher basal HH signaling activity, which, however, can be regulated by exogenous activator or inhibitor treatment, indicating an impaired endogenous repression of HH signaling and thus a compromised inhibition of HH target gene expression.

## DISCUSSION

In this study aimed at identification of the gene associated with COMA in patients who show no conclusive neuroimaging features at initial evaluation and, in particular, do not fulfill diagnostic criteria of Joubert syndrome (definite molar tooth sign), we detected heterozygous variants of *SUFU* in 15 subjects from six unrelated families of various ethnic backgrounds. Early-onset (congenital) ocular apraxia was well documented in ten patients. Eight of them showed additional neurological features including early-onset ataxia and developmental delay, with some phenotypic variability. Among the parents carrying familial *SUFU* variants, two had no history of OMA or other neurological features (II.5 family 1, I.2 family 6), one had motor and speech developmental delay as well as motor incoordination in his first years of life that regressed completely until adult age (I.1 family 2), one showed speech developmental delay during early childhood (I.2, family 1), and one shows mild OMA at adult age with unknown onset (I.1, family 5).

Of note, the neuroimaging features consistently comprise abnormalities of the superior cerebellar peduncles and the upper cerebellar vermis, but no full-blown molar tooth sign as seen in typical Joubert syndrome. The molar tooth, now considered pathognomonic for Joubert syndrome, arises from the combination of elongated, thickened, and horizontally oriented superior cerebellar peduncles, hypo-/dysplasia of the cerebellar vermis with rostral shifting of the fastigium, and an abnormally deep interpeduncular fossa at the section of the brainstem isthmus and upper pons.^16^ However, it is worth mentioning that a milder variant of the molar tooth sign was occasionally observed in patients carrying variants in certain Joubert genes including *NPHP1*, *C5orf42*, *SUFU*, and *FAM149B1*.^17–20^ These observations may suggest that the neuroimaging features in certain forms of COMA and in Joubert syndrome constitute a continuous spectrum. From our experience, a clear distinction between a definite molar tooth sign and a milder hindbrain malformation as described here is occasionally challenging, even with a technically optimal MRI investigation.

A separation of SUFU-associated COMA as reported here and Joubert syndrome based solely on clinical criteria is even more difficult and will not be possible in many cases. The clinical spectrum of Joubert syndrome encompasses a wide range of phenotypes spanning from mild variants with muscular hypotonia, ataxia, OMA, and benign developmental delay to severe forms with pronounced, sometimes progressive, multisystem disorder.^14,16^ Thus, there is considerable phenotypic overlap between these conditions.

The *SUFU* variants that we detected in the subjects described here were exclusively truncating variants. These variants are distributed over the entire protein (Fig. 3), thus making haploinsufficiency the most likely underlying pathophysiological mechanism. On a cellular level, these variants seem not to impair the occurrence, morphology or SMO localization of the primary cilium. SUFU is a major inhibitor of HH signaling, which is an evolutionary highly conserved pathway that plays an important role in embryonic development, stem cell maintenance, and tissue homeostasis.

In knockout mice with SuFu deficiency targeted to the cerebellum and some parts of the midbrain, it was demonstrated that SuFu is required for proper midhindbrain patterning, controls cerebellar patterning by regulating cell differentiation and migration, and regulates the localization and level of SHH signaling and the levels of GLIs, GLI3R in particular, and that GLI3R partially mediates SuFu functions during cerebellar morphogenesis.^21^ This prompted us to analyze the mutational impact on this pathway. Interestingly, we observed a higher basal activity of HH signaling in COMA patient–derived fibroblasts compared with control cells, supporting the hypothesis that the inhibitory function of SUFU is impaired by the identified *SUFU* variants. SAG-induced stimulation of HH signaling led to a further increase in target gene expression levels in both wild-type and patient-derived fibroblasts, whereas inhibition of HH signaling by the Smoothened inhibitor vismodegib reduced HH signaling and target gene expression to the respective basal levels. This shows that although basal activity of the HH signaling pathway is higher in COMA patient cells, pathway regulation is not impaired. Remarkably, COMA patient–derived fibroblasts show a similar fold change of HH pathway activation and repression as wild-type cells, if values were normalized to the respective basal levels.

Interestingly, germline heterozygous truncating and loss-of-function variants in *SUFU* (OMIM *607035) were observed in the basal cell nevus syndrome (BCNS, Gorlin–Goltz syndrome, OMIM 109400), a cancer-predisposing condition with variable developmental and skeletal anomalies.^22,23^ BCNS may also be associated with a heterozygous germline pathogenic variant in *PTCH1*. BCNS is mainly characterized by lamellar calcification of the falx, jaw keratocysts, palmar/plantar pits, and multiple basal cell carcinomas as major diagnostic criteria. Additionally, childhood medulloblastoma, lymphomesenteric or pleural cysts, macrocephaly, cleft lip/palate, vertebral/rib anomalies (bifid/splayed/extra ribs; bifid vertebrae), preaxial or postaxial polydactyly, ovarian/cardiac fibromas, and ocular anomalies can be observed in patients with BCNS.^24^ Among the 11 subjects with heterozygous *SUFU* variants reported here, two showed macrocephaly and one had borderline head circumference (+2.0 SD; I.1 family 2). Subject II.1 from family 3 carrying the de novo heterozygous *SUFU* variant c.479delA shows secondary, nonfamilial macrocephaly as a feature consistent with BCNS. At age six years a single digital fibroma was excised. In BCNS, ovarian and cardiac fibromas are minor diagnostic criteria, but digital fibromas were not reported, to our knowledge. Subject I.1 from family 5 shows macrocephaly (+3.0 SD) and tall stature (body height 195 cm, +2.1 SD). Parental head circumferences were not available.

Besides this, we found no other features consistent with BCNS based on physical examination and ultrasound. Thus, the condition reported here and *SUFU*-associated BCNS are likely allelic disorders, as there is currently no convincing evidence for a clinical overlap. Both, somatic and germline (heterozygous and biallelic) variants in *SUFU* were found to be associated with cerebellar medulloblastoma.^25^ A heterozygous truncating germline variant in *SUFU* was observed in familial meningioma.^26^ Given the fact that haploinsufficiency caused by truncating loss-of-function variants throughout *SUFU* is the underlying molecular mechanism in both COMA and BCNS, additional genetic and/or nongenetic modifiers must exist that drive the phenotypic expression toward a specific clinical entity. We did not observe any clusters of variants in *SUFU* or other pathway genes in our exome data, which might point toward a genetic modifier. However, the number of patients available for this analysis was limited. Consequently, larger cohorts of patients with COMA and BCNS are needed in future studies to identify these modifying factors that are determining the phenotypic outcome of heterozygous loss-of-function variants in *SUFU*.

More recently, germline biallelic pathogenic variants of *SUFU* were shown to impair SHH signaling and to be associated with Joubert syndrome type 32 (OMIM 617757).^19^ Homozygous missense variants in *SUFU* were detected in four children from two unrelated families. All subjects showed facial dysmorphism including hypertelorism; broad, depressed nasal bridge; and frontal bossing, as well as developmental delay with mild intellectual impairment, gait ataxia, and dysarthria. Three of them had postaxial polydactyly and two had global macrosomia with macrocephaly. Neuroimaging revealed cerebellar vermis hypoplasia with elongated superior cerebellar peduncles, designated by the authors as a “mild molar tooth sign.” Furthermore, polymicrogyria was present in two siblings.^19^ Interestingly, all four patients presented with ocular motor apraxia, whereas their parents, who were heterozygotes of one of the identified missense variants in *SUFU*, did not show any signs of Joubert syndrome or COMA, suggesting that heterozygous truncating variants have a more severe effect on SUFU function as a repressor of the SHH pathway activity than heterozygous hypomorphic missense variants which were identified in clinically unremarkable parents of patients with Joubert syndrome by De Mori et al.^19^

Besides these two biallelic missense variants, De Mori et al.^19^ additionally identified four individuals with heterozygous, truncating variants in *SUFU*, c.598-1G>C, c.846dupC, c.895C>T, and c.1192_1193delAA, which were either inherited from a parent or occurred de novo (Fig. 3). No details on the clinical presentation of these subjects were provided, especially not regarding the severity of their phenotype and the presence of a molar tooth sign, but in line with the individuals presented in our study, De Mori et al.^19^ observed no tumors or any signs of Gorlin syndrome in their subjects. Parents inheriting these variants were indicated as healthy. Still, based on the results of our study we observed a mild neurodevelopmental phenotype during early childhood in three parents including delayed motor and speech development without signs of OMA (individual II.3, family 1), impaired balance and poor motor coordination (individual I.1, family 2), and ongoing ophthalmological treatment from early childhood on (individual I.1, family 5). Clinical re-evaluation of the subjects with heterozygous truncating variants identified by De Mori et al.^19^ might help to determine clinical significance of these variants and evaluate the clinical spectrum of SUFU-associated variants.

Taken together, our findings indicate that heterozygous truncating germline variants in *SUFU* alter SUFU-mediated repression and increase basal activity of HH signaling pathways, resulting in COMA and a neurodevelopmental disorder with largely benign course, but some variability of clinical phenotypes. Comparing the clinical and neuroimaging features of the 15 subjects reported here with those known to occur in Joubert syndrome, we suggest addressing the clinical phenotype associated with heterozygous truncating germline variants in *SUFU* as a *forme fruste* of Joubert syndrome.

## URLs

- dbSNP, https://www.ncbi.nlm.nih.gov/snp/.
- Ensembl Genome Server, https://www.ensembl.org. gnomAD, https://gnomad.broadinstitute.org. HGMD, https://portal.biobase-international.com/.
- NCBI, https://www.ncbi.nlm.nih.gov.
- OMIM, https://www.ncbi.nlm.nih.gov/omim.
- UCSC Genome Browser, http://genome.ucsc.edu.
- UniProt, https://www.uniprot.org/. Varbank, https://varbank.ccg.uni-koeln.de/

## Supplementary information

Supplementary Information
