# Supplementary material for: Heterozygous truncating variants in SUFU cause congenital ocular motor apraxia
Source: Genet Med. 2020 Oct 7;23(2):341–51. doi: 10.1038/s41436-020-00979-w (PMC7862056; doi:10.1038/s41436-020-00979-w)
Supplement: Supplementary file 1 — Supplementary Information [file 41436_2020_979_MOESM1_ESM.pdf]

Supplementary Information

Heterozygous truncating variants in SUFU cause congenital ocular motor apraxia

Simone Schröder et al.

Material and Methods

Exome sequencing and variant screening

In family 1, trio-based exome sequencing (ES) of the affected subject III.6 and his parents (II.5 and II.6) was carried out using the NimbleGen SeqCap EZ Human Exome Library v2.0 enrichment kit (Roche) on an Illumina HiSeq4000 sequencer. In families 2 and 3, trio-based ES of the affected subjects and their parents was carried out using the Agilent SureSelect V6 (Agilent) on an Illumina HiSeq4000. ES data analysis and filtering of mapped target sequences was performed using the 'Varbank' exome analysis pipeline of the Cologne Center for Genomics (CCG, University of Cologne, Germany), and data were filtered for high-quality (coverage of more than 6 reads, a minimum quality score of 10), rare (minor allele frequency, MAF <1.0%) variants. In family 4, the coding region and flanking intronic regions were enriched using the SureSelect XT All Exon V7 in-solution technology (Agilent, Santa Clara, USA) and were sequenced using the Illumina NovaSeq system (Illumina, San Diego, USA). Illumina bcl2fastq2 was used to demultiplex sequencing reads. Adapter removal was performed with Skewer. The trimmed reads were mapped to the human reference genome (hg19) using the Burrows Wheeler Aligner and variants were called using inhouse software. Only SNVs and small indels in the coding regions and the flanking intronic regions (±8 bp) with a MAF <1.5% were evaluated. Minor allele frequencies were taken from public databases (gnomAD, dbSNP) and an in-house database. Known disease-causing variants (according to HGMD®) were evaluated in up to ±30 bp of flanking regions and up to 5% MAF. Evaluation was based on the ACMG guidelines for the interpretation of sequence variants. In the affected subject II.1 of family 5, all exons and adjacent exon-intron

boundaries of *SUFU* were analyzed by PCR and subsequent, bidirectional Sanger sequencing. In family 6, ES was performed for individual II.1 using the SureSelect V6 enrichment kit (Agilent), paired-end sequenced on an Illumina HiSeq4000 and analyzed using the pipeline of the Department of Human Genetics, Technical University of Munich, Germany, as described previously.[13]
All detected *SUFU* variants were confirmed by PCR amplification and subsequent Sanger sequencing on an independent DNA sample and tested for co-segregation within the respective families using the BigDye terminator v3.1 chemistry (Thermo Fisher Scientific) on a 3500 Genetic Analyzer (Thermo Fisher Scientific).

Cell culture and treatments

Primary dermal fibroblasts established from affected subjects III.3 (family 1), II.1 (family 2), II.1 (family 3), II.1 (family 4) and five healthy control subjects were cultured in Dulbecco's modified Eagle medium (DMEM, Gibco) supplemented with 10% fetal calf serum (FCS, Gibco) and antibiotics at 5% CO_2 and 37°C. Before treatment, cells were starved in DMEM supplemented with 0.1% FCS and antibiotics for 24 hrs. Treatment of cells was carried out with 100 nM Smoothened Agonist (SAG, Cayman Chemical), 1 µM vismodegib (Selleckchem) or a combination of both reagents for 16 hrs. SAG and vismodegib were solved in DMSO. DMSO-treated cells served as controls. Experiments were performed in biological triplicates.

Cilia formation and immunofluorescence staining

For analysis of cilia formation, fibroblasts were grown on coverslips to 90%-95% confluency. After serum starvation for 24 hrs, cells were fixed for 10 min with 4% paraformaldehyde at room temperature, permeabilized with 1x TBS containing 0.5% Triton X-100, and blocked with Tropix I-Block (Applied Biosystems) in 1x TBS containing 0.1% Triton X-100 (PBST) for

20 min. Thereafter, cells were stained with anti-acetylated tubulin (Sigma, T6793, 1:100) and anti-Smo (Abcam, ab38686, 1:500) antibodies overnight at 4°C, followed by incubation with Alexa488-conjugated anti-mouse (Jackson ImmunoResearch, 1:200) and Cy3-labeled anti-rabbit antibodies (Jackson ImmunoResearch, 1:200). Cells were mounted with ProLong Gold antifade reagent with DAPI (Thermo Fishes Scientific) and analyzed by confocal laser scanning microscopy (Olympus FLUOVIEW FV100, Olympus). Images were processed with Adobe Photoshop CS5.

Real-time quantitative PCR

Total RNA was extracted from dermal fibroblasts using TRIzol Reagent (Thermo Fisher Scientific). Synthesis of cDNA was performed using the Superscript II reverse transcriptase (Invitrogen) and random hexamer primers. Gene expression for *GLI1*, *GLI2*, *GLI3*, *HIP1,* and *PTCH1* was quantified by SYBR Green-based qRT-PCR assays on an ABI Prism HT 7900 Detection System instrument (Applied Biosystems) by using the primer pairs listed in Table S1. Data were analyzed by the standard curve method for relative quantification. Experiments were performed with two different passages per fibroblasts each analyzed in biological triplicates, which were measured in technical triplicates. Amplification of *18S r*RNA and *HPRT* were used as endogenous controls for the normalization of target gene expression.

Statistical analysis

qRT-PCR data analysis and determination of statistical differences were performed using the software GraphPad Prism 6 by nonparametric Mann–Whitney testing. Values were considered significant when $p < 0.05$.

Figure S1

Expression of Hedgehog signaling signature genes in COMA-patient derived dermal fibroblasts

A

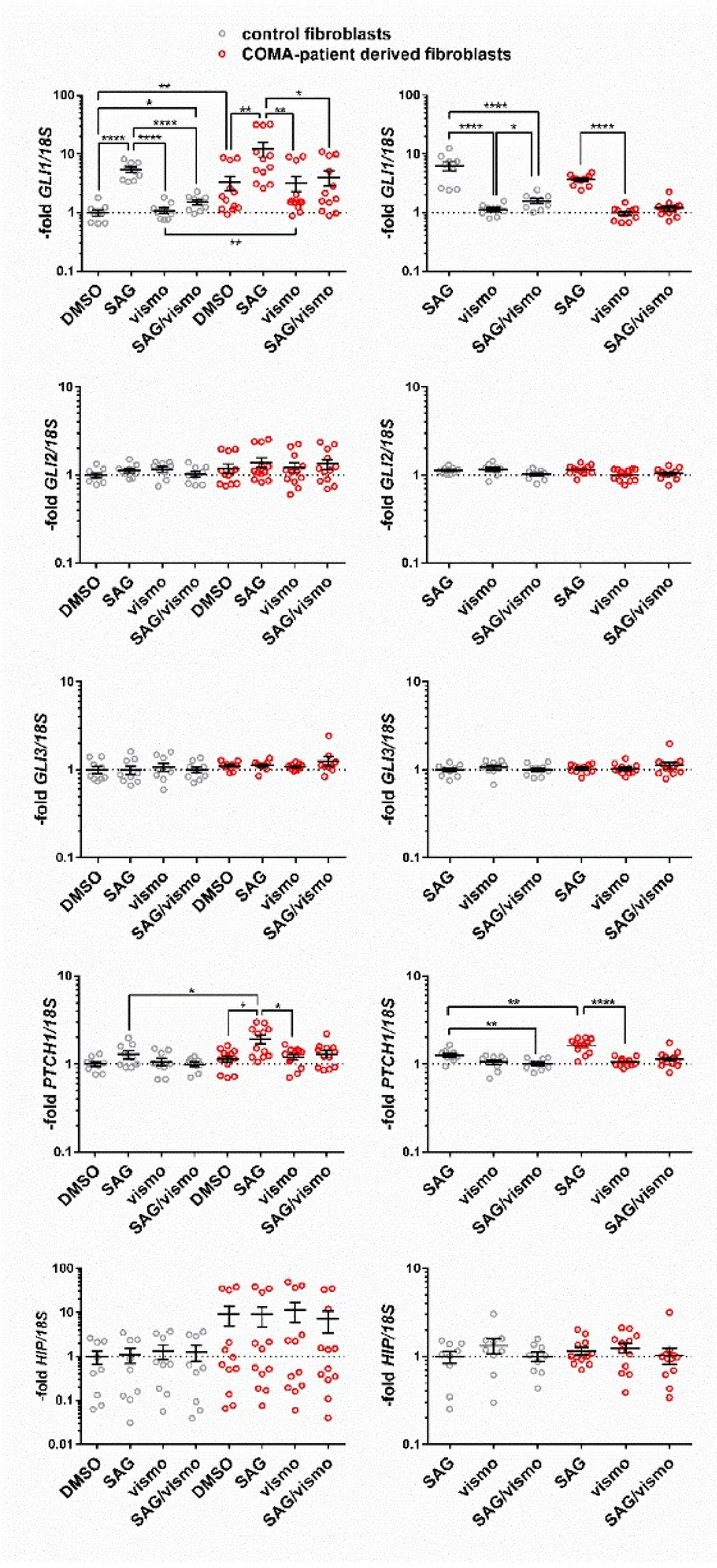

Figure S1

B

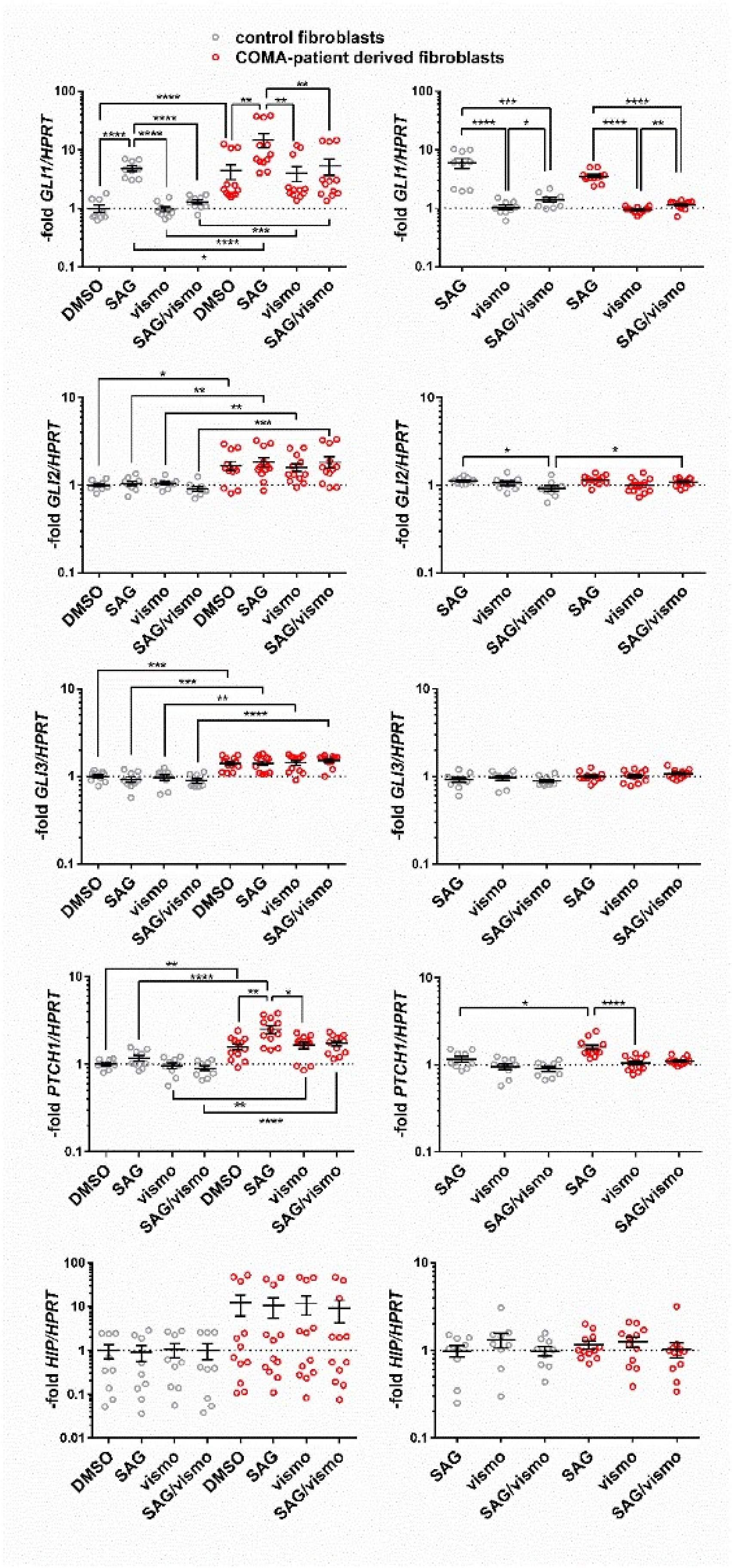
control fibroblasts
COMA-patient derived fibroblasts
-fold GLI1/HPRT
-fold GLI2/HPRT
-fold GLI3/HPRT
-fold PTCH1/HPRT
-fold HIP/HPRT
DMSO
SAG
vismo
SAG/vismo

Legend to Figure S1

Quantitative real-time PCR-based expression analyses of the Hedgehog signaling signature genes GLI1, GLI2, GLI3, HIP and PTCH1 normalized to 18S rRNA (A) or HPRT (B expression levels, respectively, of controls (N=5, grey) and COMA-patient derived fibroblasts (COMA) (N=4, red). Shown results represent data of 2 different cellular passages per fibroblast culture each analyzed in biological triplicates (grey circles) that were measured in technical triplicates. Treatment of cells was carried out with 100 nM Smoothened Agonist (SAG, Cayman Chemical), 1µM Vismodegib (Selleckchem), a combination of both reagents or DMSO only for 16 hrs. Total mean value +/- SEM of all analyzed samples are indicated in black. Significant differences were tested by nonparametric Mann-Whitney tests. *$p < 0.05$; **$p < 0.01$; ***$p < 0.001$.

Table S1 – Primers used for qRT-PCR assays

target	primer	sequence	amplicon
GLI1	forward	5‘-AGC TAC ATC AAC TCC GGC CA-3’	116 bp
	reverse	5‘-GCT GCG GCG TTC AAG AGA-3‘	
GLI2	forward	5‘-AAG CCC TTC AAG GCG CAG TA-3‘	170 bp
	reverse	5‘-TCG TGC TCA CAC ACA TAT GGC TT-3’	
GLI3	forward	5‘-GCC AGC GCA GCC CCT AT-3’	128 bp
	reverse	5‘-CGG CCT GGC TGA CAG CCT-3‘	
HIP	forward	5‘-ATG GTG GGT TGT GCT TTC CA-3‘	130 bp
	reverse	5‘-CAG AAG CAG TTG TGT TTG TGC T-3‘	
PTCH1	forward	5‘-GAG GTT GGT CAT GGT TAC ATG GA-3‘	196 bp
	reverse	5‘-TGC TGT TCT TGA CTG TGC CAC C-3‘	
HPRT	forward	5‘-TGG CGT CGT GAT TAG TGA TG-3‘	134 bp
	reverse	5‘-CGA GCA AGA CGT TCA GTC CT-3‘	
18S	forward	5‘-CGCAAATTACCCACTCCCG 3´	81 bp
	reverse	5‘-TTC CAA TTA CAG GGC CTC GAA-3’	

Congenital Ocular Motor Apraxia (COMA) questionnaire

Reporting physician: ____

Patient data

	Patient #:……..
Date of birth:	month /year _____ / __________
Sex:	□ female<br>□ male

Birth

Gestational age:	□ term<br>□ preterm (…. weeks GA)
Perinatal complications:	□ no<br>□ yes<br>If any, which? ________________________________

Family history

Other family members affected:	□ no<br>□ yes<br>If any, who? __________________________________<br>____
Consanguinity of parents:	□ no<br>□ yes

Developmental data

Age at unaided walking:	_____ years _____ months
Speech delay:	□ no<br>□ yes

Ocular findings

Ocular motor apraxia:	Onset at age _____ months
Course:	□ attenuating<br>□ normal<br>□ increasing
Jerking head movements:	□ no<br>□ yes, at age ….
Nystagmus:	□ no<br>□ yes, at age….
Involvement of vertical eye movements:	□ no<br>□ yes, at age….

Neurological findings

Ataxia:	□ no<br>□ yes, □ trunc, □ limbs
Muscular hypotonia:	□ no<br>□ yes
Cognitive development:	□ normal

	□ impaired<br><br>In case psychological test results available:<br>□ intellectual disability (IQ<70)<br>□ learning disability (IQ<85)<br>□ normal (IQ>85)
Epilepsy:	□ no<br>□ yes
Organ involvement	
Hepatic involvement:	□ no<br>□ yes
Elevated liver enzymes	□ no<br>□ yes<br>ALT: ___________ U/l<br>AST: ___________ U/l
Renal involvement:	□ no<br>□ yes
Elevated serum creatinine:	□ no<br>□ yes<br>creatinine: ______ mg/dl or ______ µmol/l
Polyuria/Polydipsia:	□ no<br>□ yes<br>□ unknown
Other clinical findings	
Irregular breathing pattern in neonatal age (i.e. apnoe, tachypnoe):	□ no<br>□ yes<br>□ unknown
Retinal anomaly (i.e. chorioidoretinal coloboma?)	□ no<br>□ yes<br>□ unknown
Dysmorphic facial features?	□ no<br>□ yes<br>If any, which? ________________________________<br>____
Skeletal features (i.e. polydactyly)?	□ no<br>□ yes<br>If any, which? ________________________________<br>____
Other clinical symptoms/abnormalities?	□ no<br>□ yes<br>If any, which? ________________________________<br>____<br>____
Previous genetic testing:	□ none<br>□ these tests were performed:<br>____<br>____<br>____<br>____

This questionnaire used in this study was published previously as a*dditional file* with reference 12:

Wente S., et al. Nosological delineation of congenital ocular motor apraxia type Cogan: an observational study. Orphanet J Rare Dis. 2016;11(1),104. doi:10.1186/s13023-016-0486-z
